# Supplementary material for: Identification of a targetable KRAS-mutant epithelial population in non-small cell lung cancer
Source: Commun Biol. 2021 Apr 14;4:370. doi: 10.1038/s42003-021-01897-6 (PMC8046784; doi:10.1038/s42003-021-01897-6)
Supplement: Supplementary file 2 — Supplementary Information [file 42003_2021_1897_MOESM2_ESM.pdf]

Supplementary Table 1. Patients information.
Patient gender, age at surgery and stage information for the 12 human NSCLCs, according to the 8th edition of the American Joint Committee on Cancer.

Tumor ID	Sex	Age at surgery (years)	Stage, AJCC 8th ed	Histology	*KRAS* Status
NSC004	M	79	pT2a N1Mx	Squamous	WT
NSC009	F	74	ypT4 N1Mx	Squamous	WT
NSC010	M	73	pT1cN0Mx	Adeno	WT
NSC016	F	61	pT4N0M1a	Adeno	Mutant
NSC018	M	83	pT1bN0Mx	Adeno	Mutant
NSC019	F	72	pT3N0M1a	Adeno	Mutant
NSC020	M	76	pT1bNxMx	Adeno	Mutant
NSC021	F	63	pT3N1Mx	Adeno	Mutant
NSC035	M	73	pT1c N0	Adeno	Mutant
NSC036	F	80	pT1b N0	Adeno	Mutant
NSC037	F	48	pT1c N0	Adeno	WT
NSC040	F	56	pT1bN2	Adeno	Mutant

Supplementary Table 2. Cell counts of kNN cluster distribution in human NSCLCs.
Size of each identified transcriptional cluster as a percentage of all profiled cells.

Cluster Number	% of Cells		FDR
	KRAS-Mutant	*KRAS*-WT	
1	3.55	0.59	$3.68x10^{-89}$
2	6.18	2.26	$2.92x10^{-74}$
3	2.08	1.70	$6.50x10^{-2}$
4	22.73	5.86	0.00
5	3.24	29.69	0.00
6	15.06	13.56	$9.95x10^{-54}$
7	8.19	3.49	$1.08x10^{-72}$
8	16.64	14.45	$5.36x10^{-08}$
9	3.13	1.53	$4.00x10^{-31}$
10	2.77	0.13	$2.07x10^{-116}$
11	1.90	3.71	$8.22x10^{-27}$
12	2.24	0.98	$4.94x10^{-33}$
13	3.73	4.78	$6.04x10^{-06}$
14	4.44	8.59	$6.51x10^{-75}$
15	4.10	8.68	$2.36x10^{-92}$

Supplementary Table 3. Gene listing of the uniquely identifying per cluster marker genes for healthy murine samples.
Tabular listing of the marker genes depicted in Supplementary Figure 2a in corresponding order.

Top Marker Genes Per Cluster in Healthy Tissue							
1. Rtn3	31. Ear2	61. Rpl9-ps6	91. C1qb	121. Bgn	151. Tmem100	181. Ager	211. Chchd10
2. Ccnd2	32. Ear1	62. Rpl41	92. C1qa	122. Inmt	152. Ly6a	182. Tmem63b	212. Cyp2f2
3. Rp23-27H19.7	33. Eif4a1	63. Gm10076	93. Cd83	123. Gsn	153. Pecam1	183. Ptprf	213. Dynlrb2
4.Tmsb4x	34. Emp3	64. Rps23-ps1	94. H2-Ab1	124. Serping1	154. Ace	184. Alcam	214. Ccdc153
5. Alox5ap	35. Vim	65. Rps15a	95. H2-Eb1	125. C1s1	155. Ramp2	185. Gpx4	215. Sec14l3
6. Cebpb	36. Psap	66. Rps11	96. Cd74	126. Clec3b	156. Calcrl	186. Dstn	216. Cbr2
7. Fcer1g	37. Fth1	67. Rplp0	97. H2-Aa	127. Col3a1	157. Cdh5	187. Myl6	217. Wfdc2
8.Tyrobp	38. Ftl1	68. Gm8730	98. Jund	128. Col1a2	158. Adgrf5	188. Sod1	218. Aldh3b1
9. Plek	39. Ftl1-ps1	69. Rpl10a-ps1	99.Hsp90aa1	129. Col1a1	159. Cd36	189. Tuba1a	219. Tppp3
10. Cxcl2	40. Ccr7	70. Tmsb10	100. Hspa1a	130. Sparc	160. Hpgd	190. Gstm1	220. Ctsh
11. Arpc2	41. Ms4a4b	71. Gm10275	101. Junb	131. Fstl1	161. Epas1	191. Igfbp5	221. Npc2
12. Cd9	42. Vps37b	72. Rps28	102. Tmem176b	132. Plac9b	162. Tspan7	192. Maged1	222. Scd1
13. Iqgap1	43. Satb1	73. Gm6472	103. Selenbp1	133. Plac9a	163. Ppp1r2	193. Gm4076	223. Cxcl15
14. Anxa2	44. Ets1	74. Tpt1	104. Aldh1a1	134. Hnrnpk	164. Rbms1	194. Gm11410	224. Sftpb
15. Laptm5	45. H2-K1	75. Rps24	105. Npnt	135. Dazap2	165. Tmem204	195. mt-Co1	225. Sftpc
16. Cd44	46. H2-Q7	76. Mxd1	106. Limch1	136. Myl12a	166. Marcks	196. mt-Nd2	226. Slc34a2
17. Mrc1	47. Gm6274	77. Slc16a3	107. Sh3bgrl	137. Cav2	167. Selenok	197. mt-Co3	227. Pcbp2
18. Plin2	48. Rpl21	78. Hdc	108. Cst3	138. Smad6	168. Il6st	198. mt-Nd4	228. Coq5
19. Lgals3	49. Gm16288	79. Ptprc	109 Plpp3	139. Foxf1	169. Antxr1	199. mt-Cytb	229. Yars
20. Mpeg1	50. Rps25-ps1	80. Cd53	110. Prelp	140. Sptbn1	170. Rsrp1	200. mt-Atp6	230. Pop4
21. Ctss	51. Rps25	81. Stk17b	111. Fmo2	141. Selenop	171. Unc13d	201. Nudc	231. Tpr
22. Cybb	52. Rp23-263B18.4	82. Srgn	112. Gpx3	142. Icam2	172. Slco3a1	202. Calm1	232. Eif3c
23. Lyz2	53. Rpl37rt	83. Btg1	113. Crispld2	143. Aqp1	173. Col4a2	203. Pcp4l1	233. Rbm3
24. Lyz1	54. Rpl10	84. Pim1	114. Sparcl1	144. Podxl	174. Bcam	204. Cd24a	234. Pcbp1
25. Krt79	55. Rp23-235E15.1	85. Il1b	115. Sod3	145. Fmo1	175. Crip2	205. Cfap126	235. Ran
26. Lpl	56. Rps29	86. Mcl1	116. Cd302	146. Thbd	176. Vamp8	206. Cyp2s1	
27.Ctsd	57. Rps14	87. Actg1	117. Adamts2	147. Clec14a	177. Pdpn	207. Cdhr3	
28. Plet1	58. Rps27	88. Kctd12	118. Rnase4	148. Bmpr2	178. Gprc5a	208. Tubb4b	
29. Ccl6	59. Rps27rt	89. Cd14	119. Ltbp4	149. Ehd4	179. Scd2	209. BC051019	
30. Chil3	60. Rpl9	90. C1qc	120. Mgp	150. Egfl7	180. Cldn18	210. Rsph1	

Supplementary Table 4. Gene listing of the uniquely identifying per cluster marker genes for murine KP tumor samples.
Tabular listing of the marker genes depicted in Supplementary Figure 2b in corresponding order.

Top Marker Genes Per Cluster in Tumor Tissue							
1. Ly6e	31. Tpt1	61. Col1a2	91. Arhgef5	121. Cldn3	151. Cst3	181. Mmp9	211. Ccl6
2. Ly6a	32. Eef1a1	62. Col1a1	92. Lsr	122. Ager	152. H2-Ab1	182. Clec4e	212. Chil3
3. Ly6c1	33. Rps26	63. Mgp	93. Foxq1	123. Cldn18	153. Cd74	183. Il1rn	213. Arg1
4. Ly6c2	34. Gm8730	64. Serping1	94. mt-Co1	124. Lgi3	154. H2-Aa	184. Ccrl2	214. Fn1
5. Fkbp1a	35. Rplp0	65. Dlk1	95. Tsc22d1	125. Wfdc2	155. H2-Eb1	185. Naaa	215. Calm1
6. Hspb1	36. Rpl13a	66. Meg3	96. Spint2	126. Npc2	156. H2-K1	186. Car4	216. Tmsb10
7. Serpinh1	37. Rps2	67. Fmo2	97. Cystm1	127. Slc34a2	157. Odc1	187. Lgals1	217. Gm9844
8. Sparcl1	38. Rps18	68. Gdpd2	98. Clu	128. Sftpc	158. Crem	188. Vim	218. Ergic1
9. Igfbp7	39. Gm10275	69. Armt1	99. Krt7	129. Cxcl15	159. Kctd12	189. B2m	219. Pkm
10. Sparc	40. Rpl32	70. Stk38	100. Krt8	130. Sftpb	160. Bcl2a1b	190. Gns	220. Spp1
11. Col4a1	41. Rplp1	71. Dynll1	101. Krt18	131. Sftpd	161. Tnfaip3	191, Tcf7l2	221. Cd9
12. Col4a2	42. Rps29	72. Tuba1a	102. Mt1	132. Lpcat1	162. Cxcl2	192. Fabp5	222. Anxa2
13. Nrp1	43. Rps27	73. Tubb4b	103. Prdx1	133. Napsa	163. Nfkbia	193. Gpnmb	
14. Cd93	44. Rps27rt	74. S100a11	104. Gstm1	134. Hc	164. Pim1	194. Plin2	
15. Rtl8a	45. Rpl10	75. Cd24a	105. Cd63	135. Scd1	165. Il1b	195. Lyz2	
16. Rtl8c	46. Gm4613	76. Cxcl17	106. Tns1	136. Rasl11a	166. Srgn	196. Lyz1	
17. Eng	47. Rpl41	77. Aldh1a1	107. Txnip	137. Mettl7a1	167. Rac2	197. Cybb	
18. Ctla2a	48. Rps14	78. Tppp3	108. Hsp90aa1	138. Cbr2	168. Cytip	198. Ctsd	
19. Esam	49. Rps19	79. Ccdc153	109. Ubb	139. Cyp2f2	169. Cd53	199. Ftl1	
20. Kdr	50. Rpl38	80. Foxj1	110. Jun	140. Mmp12	170. Fcer1g	200. Ftl1-ps1	
21. Cd34	51. Rack1	81. Scara3	111. Fos	141. Fcgr2b	171. Fth1	201. Laptm5	
22. Aqp1	52. Gm15427	82. Qsox1	112. Tmem176b	142. Ly86	172. Tyrobp	202. Mpeg1	
23. Egfl7	53. Rps28	83. Gsto1	113. Gnas	143. Csf1r	173. Plek	203. Ctss	
24. Plvap	54. Thy1	84. Fxyd3	114. Emp2	144. Apoe	174. Cebpb	204. Psap	
25. Pecam1	55. Timp3	85. Nrn1	115. Spint1	145. C1qc	175. Lilrb4a	205. Sirpa	
26. Cdh5	56. Gpx3	86. Prss22	116. Uhrf1bp1l	146. C1qb	176. Nfe2l2	206. Atp6v0d2	
27. Rpsa-ps1	57. Bgn	87. S100a6	117. Nedd4l	147. C1qa	177. Btg2	207. Plet1	
28. Rps23	58. Fstl1	88. Ctse	118. Nkx2-1	148. Cyth4	178. G0s2	208. Ear2	
29. Rps24	59. Col5a2	89. Krt20	119. Egfl6	149. Cd52	179. S100a9	209. Ear1	
30. Gm6472	60. Col3a1	90. Cdkn2a	120. Ctsh	150. Mgl2	180. Marcksl1	210. Lpl	

Supplementary Table 5. Cell counts of kNN cluster distribution in murine tissues.
Size of each identified transcriptional cluster as a percentage of all profiled cells.

Cluster Number	% Cells		FDR
	Healthy Tissue	Tumor Tissue	
1	4.1	5.6	2.46×10^{-03}
2	0.1	3.7	4.22×10^{-56}
3	31.2	4.8	0.00
4	2.5	1.0	4.42×10^{-10}
5	11.1	16.3	4.39×10^{-15}
6	9.6	9.7	1.00
7	23.5	2.7	1.47×10^{-288}
8	2.8	12.8	4.92×10^{-95}
9	0.5	0.9	6.58×10^{-01}
10	0.2	11.8	1.36×10^{-192}
11	11.3	2.2	1.12×10^{-96}
12	2.6	10.8	1.05×10^{-72}
13	0.3	17.8	1.22×10^{-289}

Supplementary Table 6. Cell counts of annotated subpopulations in murine tissues.
Size of each identified cell type as a percentage of all profiled cells.

Cell Type	% Cells		FDR
	Healthy Tissue	Tumor Tissue	
Alveolar Bipotent Progenitor	0.1	2.3	2.62×10^{-32}
Alveolar Type I	1.9	2.2	1.00
Alveolar Type II	1.2	31.7	0.00
Basal Epithelial Cell	0.0	2.0	4.64×10^{-33}
Basophil	0.0	0.0	1.00
B Cell	1.8	0.5	7.11×10^{-10}
Ciliated Cell	2.7	1.0	1.26×10^{-11}
Club Cell	0.1	1.4	2.32×10^{-15}
Dendritic Cell	3.4	3.0	1.00
Dividing Cell	0.0	0.3	5.04×10^{-04}
Endothelial Cell	30.7	5.8	6.86×10^{-299}
Eosinophil	0.0	0.0	1.00
Epithelial Cell	0.1	5.0	7.77×10^{-78}
Erythroblast	0.2	0.0	4.85×10^{-03}
Fibroblast	19.3	1.3	6.46×10^{-275}
Innate Lymphoid Cell	1.0	0.6	0.46
Macrophage	18.9	33.7	3.53×10^{-74}
Mast Cell	0.0	0.0	1.00
Monocyte	3.6	0.6	2.15×10^{-33}
Neutrophil	1.3	6.6	5.21×10^{-50}
Natural Killer Cell	0.6	0.0	1.83×10^{-09}
Natural Killer T Cell	1.2	0.5	5.60×10^{-03}
Stromal Cell	6.5	1.0	2.25×10^{-60}
T Cell	5.4	0.4	1.60×10^{-73}
Unknown	0.0	0.2	0.0450

Supplementary Table 7. 50 Highest/Lowest Genes shown in the heatmap depicted in Figure 3a.
Tabular listing of the 50 highest/lowest expressed genes shown in Figure 3a. Genes are listed in the corresponding order found in the heatmap. Table lists for each gene the corresponding log fold change (logFC) and the Benjamini-Hochmberg adjusted False Discovery Rate (FDR) for each gene.

Highest 50			
Genes	LogFC	P Value	FDR
Clu	4.31	0	0
S100a6	3.00	$3.58x10^{-218}$	$3.07x10^{-215}$
Tff1	5.81	$2.35x10^{-121}$	$5.11x10^{-119}$
Ltf	5.75	$8.22x10^{-187}$	$5.18x10^{-184}$
Gsto1	3.99	0	0
Dmbt1	4.67	$5.01x10^{-238}$	$6.30x10^{-235}$
Krt7	2.71	0	0
Tff2	3.48	$3.73x10^{-27}$	$8.34x10^{-26}$
Ldha	1.76	$5.12x10^{-243}$	$7.43x10^{-240}$
Ctse	3.75	0	0
Spp1	5.03	$8.83x10^{-225}$	$8.34x10^{-222}$
Anxa2	2.12	$3.29x10^{-266}$	$6.21x10^{-263}$
Hspa1b	1.76	$5.57x10^{-112}$	$1.03x10^{-109}$
Krt8	1.65	$1.06x10^{-176}$	$5.89x10^{-174}$
Qsox1	2.93	$4.03x10^{-298}$	0
Pkm	2.10	$2.42x10^{-306}$	$5.73x10^{-303}$
Tsc22d1	1.71	$2.26x10^{-239}$	$3.05x10^{-236}$
Ybx1	1.56	$5.63x10^{-235}$	$6.26x10^{-232}$
S100a11	1.44	$3.67x10^{-180}$	$2.10x10^{-177}$
Cd9	1.51	$1.31x10^{-202}$	$1.08x10^{-199}$
Lgals3	2.06	$2.62x10^{-135}$	$7.72x10^{-133}$
Areg	1.98	$2.62x10^{-115}$	$5.10x10^{-113}$
Tpt1	1.13	$9.48x10^{-224}$	$8.53x10^{-221}$
Gm8730	1.27	$4.44x10^{-181}$	$2.62x10^{-178}$
Fxyd3	3.65	0	0
Onecut2	3.20	$1.89x10^{-245}$	$2.97x10^{-242}$
F3	2.91	$6.69x10^{-231}$	$7.02x10^{-228}$
Gm9843	1.28	$4.17x10^{-202}$	$3.28x10^{-199}$
Krt18	1.64	$6.39x10^{-199}$	$4.47x10^{-196}$
Hspa1a	1.52	$7.62x10^{-72}$	$6.79x10^{-70}$
Ly6e	1.39	$1.36x10^{-157}$	$5.71x10^{-155}$
Anxa1	2.24	$2.45x10^{-186}$	$1.49x10^{-183}$
Gm15427	1.11	$1.25x10^{-162}$	$5.77x10^{-160}$
Ly6a	3.12	$5.14x10^{-168}$	$2.62x10^{-165}$
Anxa3	1.86	$2.64x10^{-191}$	$1.78x10^{-188}$
Calr	1.31	$1.97x10^{-137}$	$6.10x10^{-135}$
Rplp0	1.22	$5.98x10^{-128}$	$1.53x10^{-125}$
Rplp1	1.02	$8.14x10^{-124}$	$1.87x10^{-121}$
Rpl18-ps1	1.11	$2.33x10^{-141}$	$7.86x10^{-139}$
Hsp90ab1	0.93	$9.15x10^{-122}$	$2.03x10^{-119}$
Krt19	2.80	$4.66x10^{-284}$	$9.78x10^{-281}$
Gng5	1.26	$2.37x10^{-155}$	$9.53x10^{-153}$
Dpcr1	1.99	$2.59x10^{-61}$	$1.80x10^{-59}$
Tpi1	1.53	$1.32x10^{-145}$	$4.71x10^{-143}$
Kcne3	1.00	$9.56x10^{-39}$	$3.37x10^{-37}$
mt-Nd2	1.07	$5.54x10^{-124}$	$1.29x10^{-121}$
Ezr	1.37	$2.31x10^{-121}$	$5.08x10^{-119}$
Rps28	0.93	$3.90x10^{-133}$	$1.08x10^{-130}$
Rps2-ps10	1.04	$1.54x10^{-89}$	$2.01x10^{-87}$
Rps2	1.01	$2.55x10^{-94}$	$3.62x10^{-92}$

Lowest 50			
Genes	LogFC	P Value	FDR
Mlc1	-1.13	$2.00x10^{-37}$	$6.66x10^{-36}$
Dnajb4	-0.82	$6.12x10^{-18}$	$8.58x10^{-17}$
Lpcat1	-1.94	$3.44x10^{-108}$	$6.03x10^{-106}$
Fmo2	-2.20	$2.15x10^{-83}$	$2.47x10^{-81}$
Rps3a3	-1.34	$5.18x10^{-36}$	$1.62x10^{-34}$
Lrg1	-1.58	$2.89x10^{-29}$	$7.09x10^{-28}$
Porcn	-1.11	$1.03x10^{-20}$	$1.71x10^{-19}$
Prrg3	-0.81	$2.34x10^{-17}$	$3.15x10^{-16}$
Msrb2	-0.49	$2.61x10^{-12}$	$2.42x10^{-11}$
Lrrc26	0.58	$1.68x10^{-21}$	$2.89x10^{-20}$
Nkd1	-1.28	$1.37x10^{-41}$	$5.26x10^{-40}$
Elovl1	-1.41	$1.89x10^{-55}$	$1.11x10^{-53}$
Mylk	-0.99	$2.80x10^{-26}$	$6.09x10^{-25}$
Rnase4	-1.71	$3.30x10^{-93}$	$4.62x10^{-91}$
Gm14137	0.26	$2.89x10^{-09}$	$2.03x10^{-08}$
C6	-0.89	$8.51x10^{-26}$	$1.81x10^{-24}$
Acot1	-0.93	$4.26x10^{-31}$	$1.12x10^{-29}$
Fam83e	0.62	$4.70x10^{-21}$	$7.93x10^{-20}$
Anpep	-0.58	$2.17x10^{-15}$	$2.55x10^{-14}$
Kcnj15	-1.79	$5.60x10^{-75}$	$5.34x10^{-73}$
Cldn18	-1.12	$7.68x10^{-51}$	$3.91x10^{-49}$
Scnn1g	-0.44	$2.02x10^{-10}$	$1.58x10^{-09}$
Napsa	-1.57	$6.40x10^{-48}$	$2.92x10^{-46}$
Lbh	1.14	$5.36x10^{-51}$	$2.74x10^{-49}$
Ms4a8a	0.35	$3.76x10^{-12}$	$3.44x10^{-11}$
Cacna1h	0.57	$2.59x10^{-16}$	$3.27x10^{-15}$
Lamp3	-1.79	$1.13x10^{-65}$	$8.58x10^{-64}$
Cyp2f2	-1.26	$4.34x10^{-15}$	$4.94x10^{-14}$
Gem	-1.17	$1.06x10^{-35}$	$3.27x10^{-34}$
Gm8113	-0.80	$7.94x10^{-16}$	$9.67x10^{-15}$
Pf4	-1.70	$5.54x10^{-49}$	$2.65x10^{-47}$
Efs	0.31	$5.52x10^{-09}$	$3.78x10^{-08}$
Lyz1	-1.86	$2.24x10^{-55}$	$1.31x10^{-53}$
Mettl7a1	-1.65	$3.78x10^{-75}$	$3.63x10^{-73}$
Hc	-1.90	$7.35x10^{-56}$	$4.42x10^{-54}$
Lrmp	0.59	$2.66x10^{-23}$	$5.01x10^{-22}$
Ager	-2.82	$3.45x10^{-230}$	$3.43x10^{-227}$
Scgb3a1	-2.88	$3.50x10^{-13}$	$3.49x10^{-12}$
Npc2	-0.95	$2.46x10^{-68}$	$1.98x10^{-66}$
Cyp2b10	-0.85	$1.18x10^{-13}$	$1.22x10^{-12}$
H2-Aa	-2.36	$7.14x10^{-126}$	$1.80x10^{-123}$
Cxcl15	-1.66	$1.35x10^{-63}$	$9.87x10^{-62}$
Cd74	-1.78	$1.32x10^{-125}$	$3.19x10^{-123}$
Gc	-1.23	$4.32x10^{-20}$	$6.87x10^{-19}$
Cbr2	-4.01	$1.22x10^{-138}$	$3.97x10^{-136}$
Sftpa1	-2.04	$6.42x10^{-67}$	$4.99x10^{-65}$
Dlk1	-3.63	$3.20x10^{-65}$	$2.42x10^{-63}$
Bpifa1	-2.59	$9.56x10^{-33}$	$2.68x10^{-31}$
Lyz2	-3.31	$6.38x10^{-129}$	$1.67x10^{-126}$
Sftpc	-2.87	$3.83x10^{-124}$	$9.05x10^{-122}$

Supplementary Table 8. RelA/p65 pathway. Following differential gene expression between murine and human C10 versus the other epithelial clusters, EnrichR software indicates RelA/p65 pathway as enriched in both species. Table shows P values, FDR, Odds Ratio and Combined Score.

RELA_24523406_ChIP-Seq_FIBROSARCOMA_Human	P-value	FDR	Odds Ratio	Combined Score
Mouse C10 Vs Other Epithelial Clusters	1.51×10^{-4}	0.01	2.55	22.46
Human C10 Vs Other Epithelial Clusters	6.94×10^{-4}	0.03	2.08	15.14

Supplementary Table 9. Cross-tabs comparing Human and Murine clusters/subpopulations. Tables show (a) predominant cell populations present in each murine and/or human cluster and (b) cell types across both species.

a

Human Lung Cancer		KP Lung Cancer
**Cluster #**	**Predominant Cell Population**	**Predominant Cell Population**
**C1**	Neutrophils	Macrophages
**C2**	Epithelial Cells	AT2-Like cells
**C3**	Natural Killer Cells	Endothelial Cells
**C4**	T cells	Ciliated Cells
**C5**	Epithelial Cells	Macrophages
**C6**	Macrophges	Monocytes/Neutrophils/Dendritic Cells
**C7**	B Cells/T Cells	Fibroblasts/Stromal Cells
**C8**	B Cells/T Cells	AT2-Like cells/AT1-Like cells
**C9**	B Cells	AT2-Like cells
**C10**	Epithelial Cells	Epithelial cells
**C11**	Monocytes	B cells/T Cells/NK/NKT/ILC
**C12**	B Cells	Macrophages/Dendritic Cells
**C13**	Fibroblasts/Endothelial Cells	AT2-Like cells
**C14**	Dendritic Cells/Monocytes/Macrophages	
**C15**	Dendritic Cells/Monocytes/Macrophages	

b

Main Cell Populations	Human Lung Cancer	KP Lung Cancer
Neutrophils	C1	C6
Epithelial Cells	C2, C5, C10	C2, C4, C8, C9, C10, C13
Natural Killer Cells	C3	C11
T cells	C4	C11
Macrophages	C6	C1, C5, C12
B Cells	C7, C8, C9, C12	C11
Monocytes	C11	C6
Fibroblasts	C13	C7
Endothelial Cells	C13	C3
Dendritic cells	C14, C15	C6, C12

Supplementary Figure 1. SPRING plots showing patients' distribution in KRAS-mutant and KRAS-WT samples.

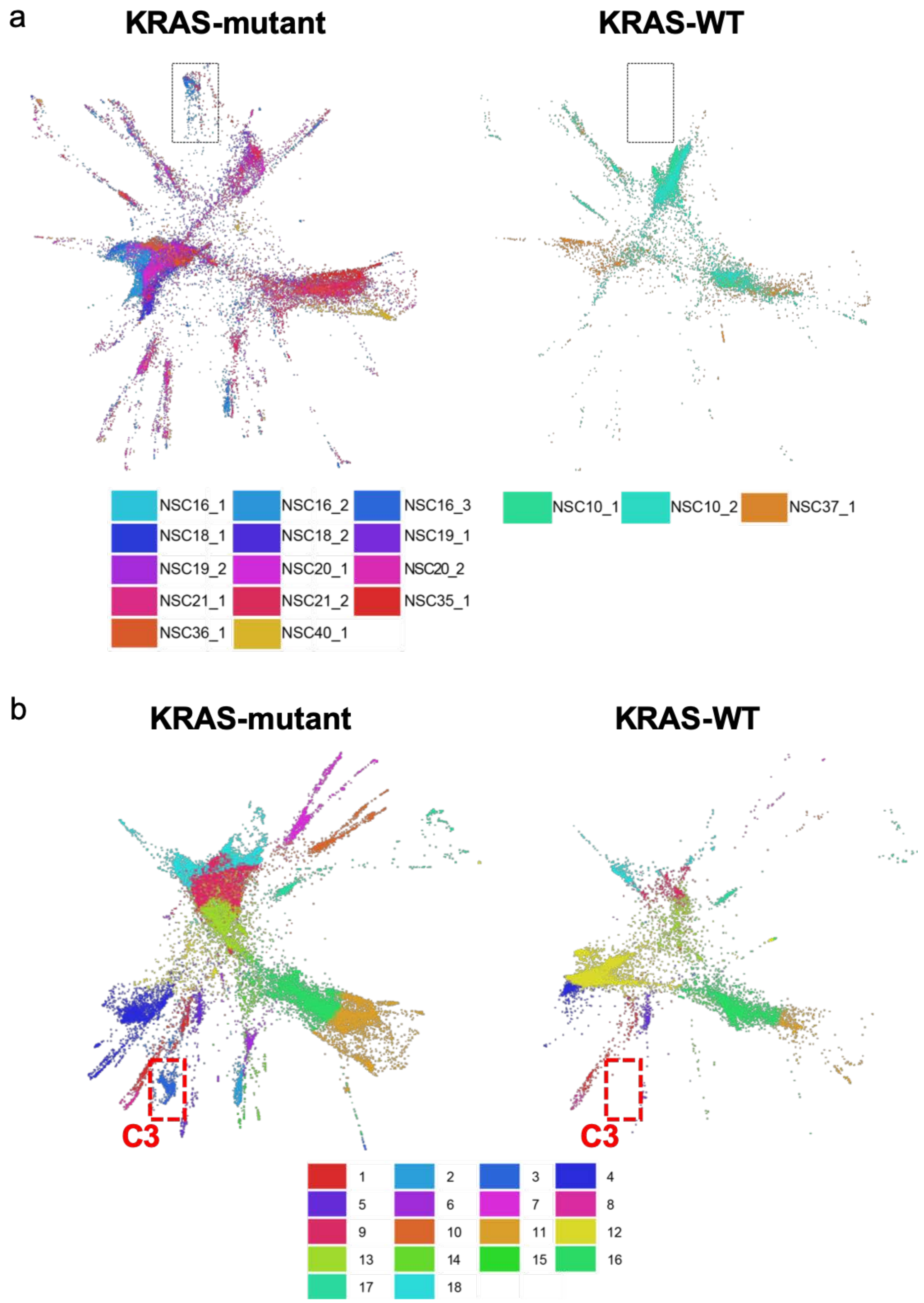

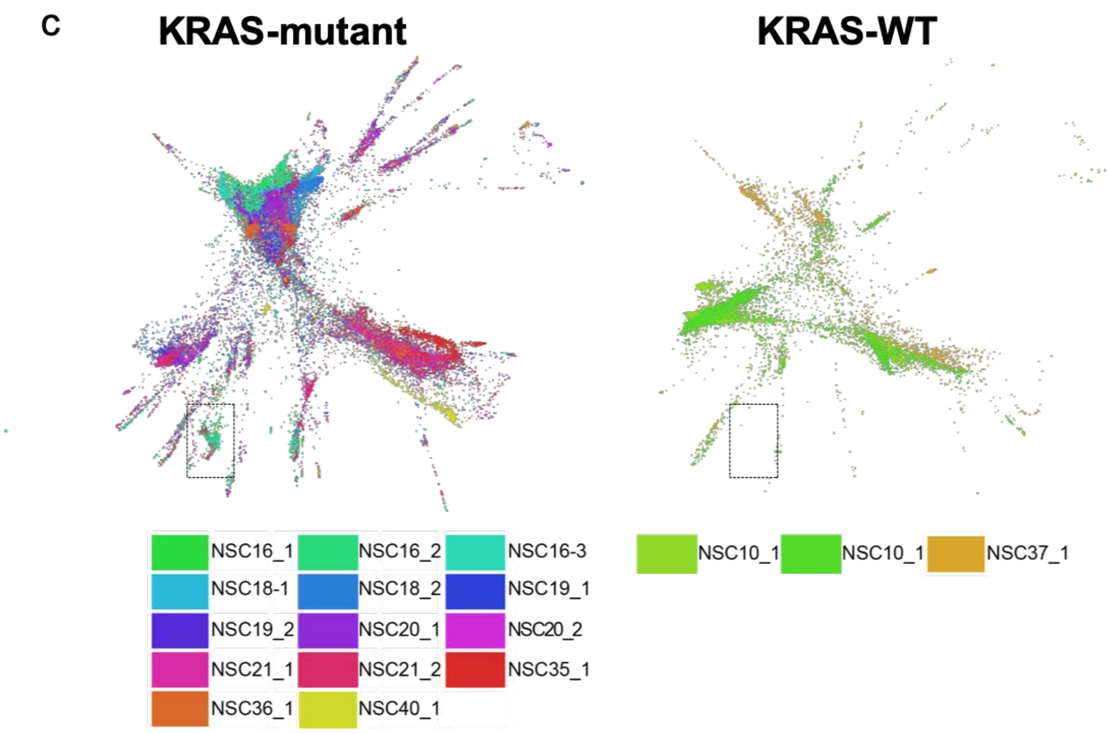

a SPRING plot shows patients' contribution to C10 (black dotted box), enriched in KRAS-mutant adenocarcinomas. **b** Also SPRING plots generated only on the adenocarcinoma subset display a unique cluster (cluster C3), highlighted by the red dotted box, which is enriched in KRAS-mutant patients (left panel) as compared to the KRAS-WT patients (right panel) ($p = 7.52 \times 10^{-90}$).
c SPRING plot shows patients' contribution to C3 (black dotted box) enriched in KRAS-mutant samples.

Supplementary Figure 2. Characterization of murine tumor clusters

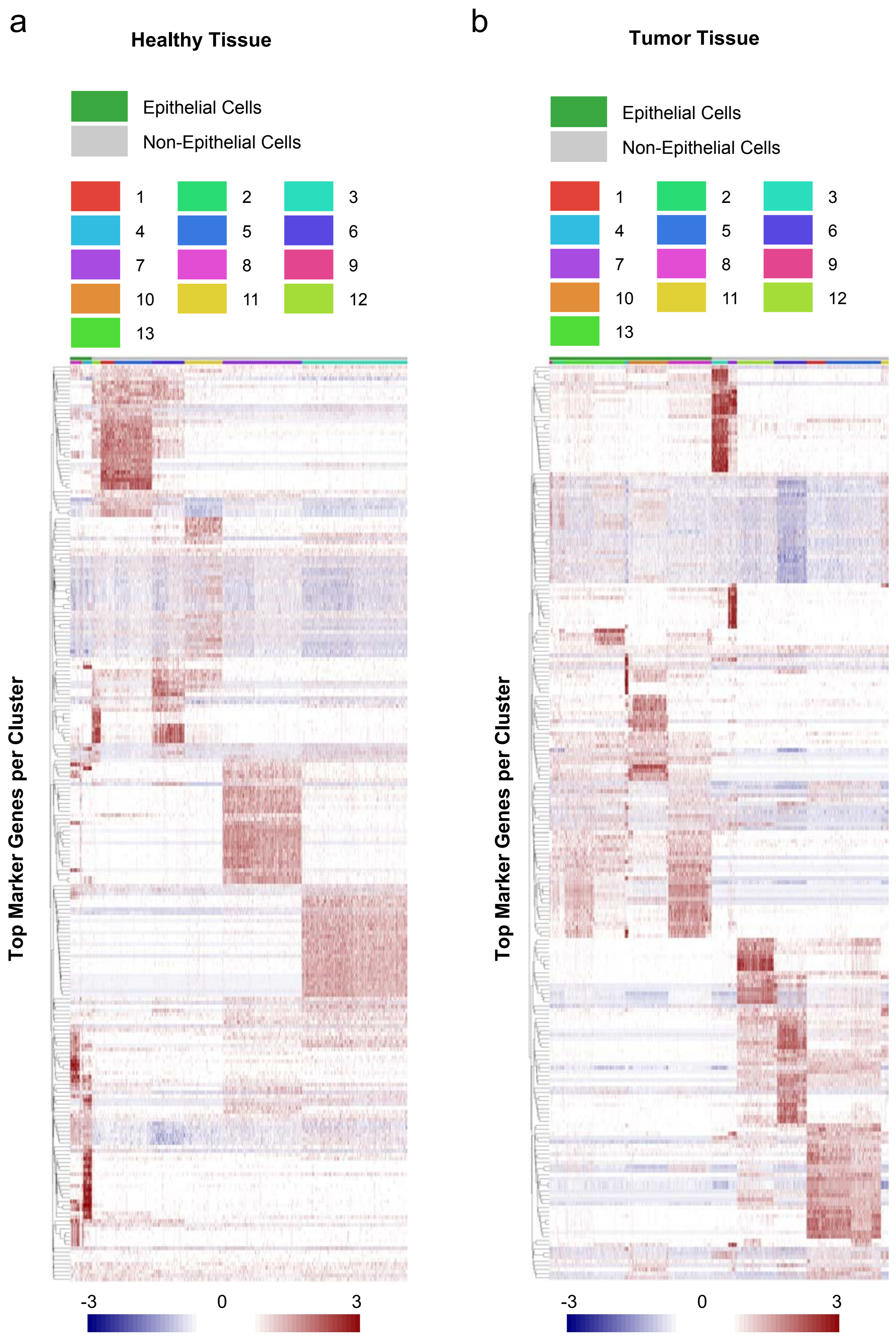

c

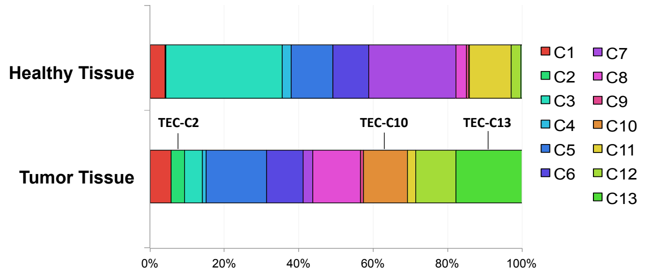
Healthy Tissue
Tumor Tissue
TEC-C2
TEC-C10
TEC-C13
0%
20%
40%
60%
80%
100%
C1
C2
C3
C4
C5
C6
C7
C8
C9
C10
C11
C12
C13

d

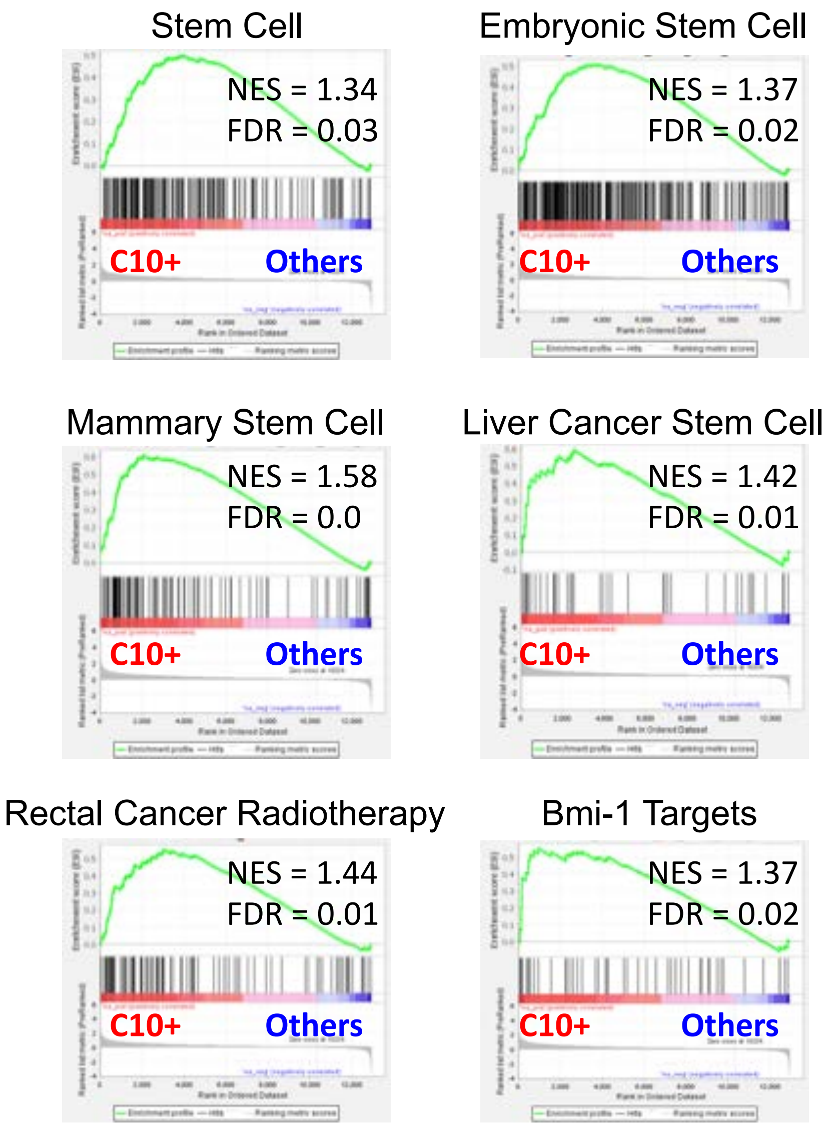
Stem Cell
NES = 1.34
FDR = 0.03
C10+
Others
Embryonic Stem Cell
NES = 1.37
FDR = 0.02
C10+
Others
Mammary Stem Cell
NES = 1.58
FDR = 0.0
C10+
Others
Liver Cancer Stem Cell
NES = 1.42
FDR = 0.01
C10+
Others
Rectal Cancer Radiotherapy
NES = 1.44
FDR = 0.01
C10+
Others
Bmi-1 Targets
NES = 1.37
FDR = 0.02
C10+
Others

Heatmaps show unique genes in **a** healthy tissue and **b** tumor tissue from the 25 highest expressed genes per cluster. The genes depicted herein are listed in Supplementary Tables 3 and 4, in the corresponding order. **c** Comparison of clusters distribution in healthy and tumor tissue shows 3 specific tumor clusters (TEC-C2, TEC-C10, TEC-C13). **d** GSEA of differentially expressed genes in C10, as compared to all other tumor epithelial clusters, shows enrichment of Stem Cell (NES=1.34, FDR=0.03), Embryonic Stem Cell (NES=1.37, FDR=0.02), Mammary Stem Cell (NES=1.58, FDR=0.0), and Liver Cancer Stem Cell (NES=1.42, FDR=0.01) signatures, as well as Rectal Cancer Radiotherapy (NES=1.44, FDR=0.01) and Bmi-1 target (NES=1.37, FDR=0.02) genes.

Supplementary Figure 3. The conserved mutant *KRAS*-associated subpopulation showing enrichment for KRAS-aggressive pathways and positivity for the oncogene *BMI*-1 in KP tumors and *KRAS* Mutant Patients.

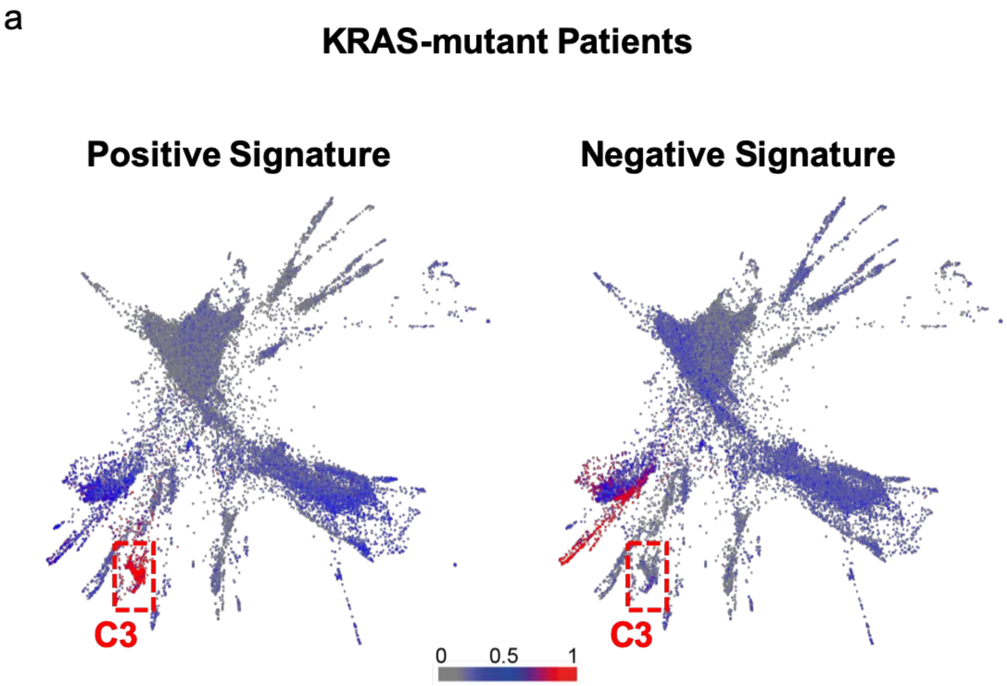

b

Murine EIF2 Signaling

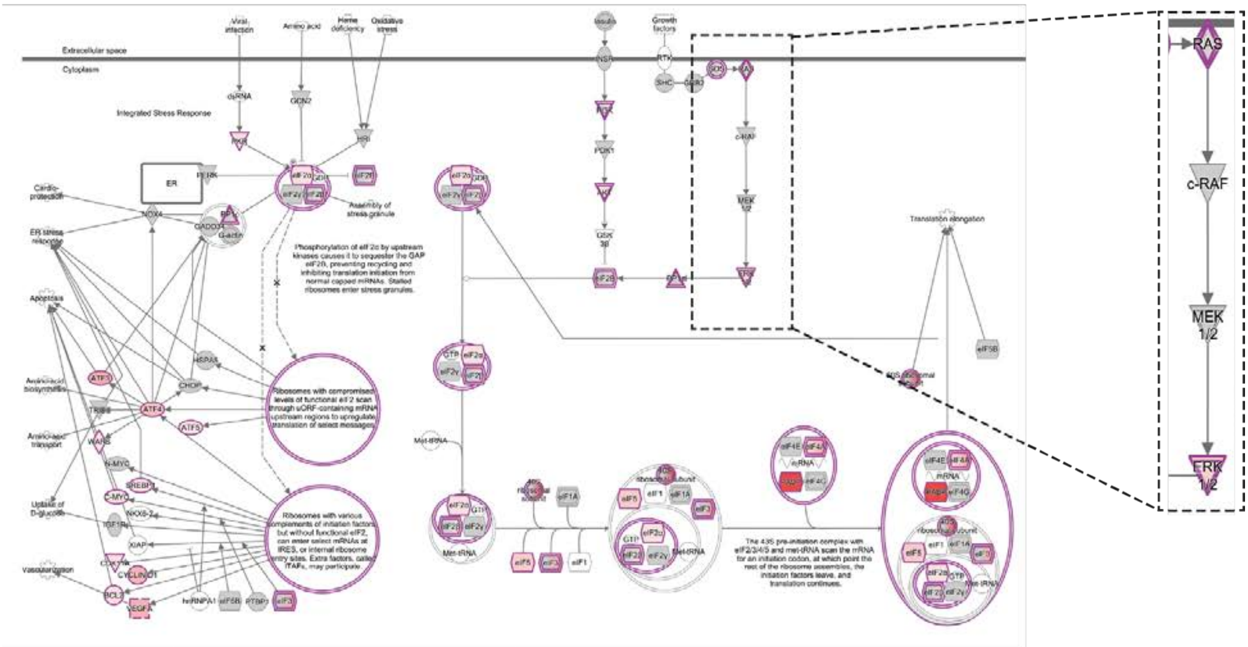

Human EIF2 Signaling

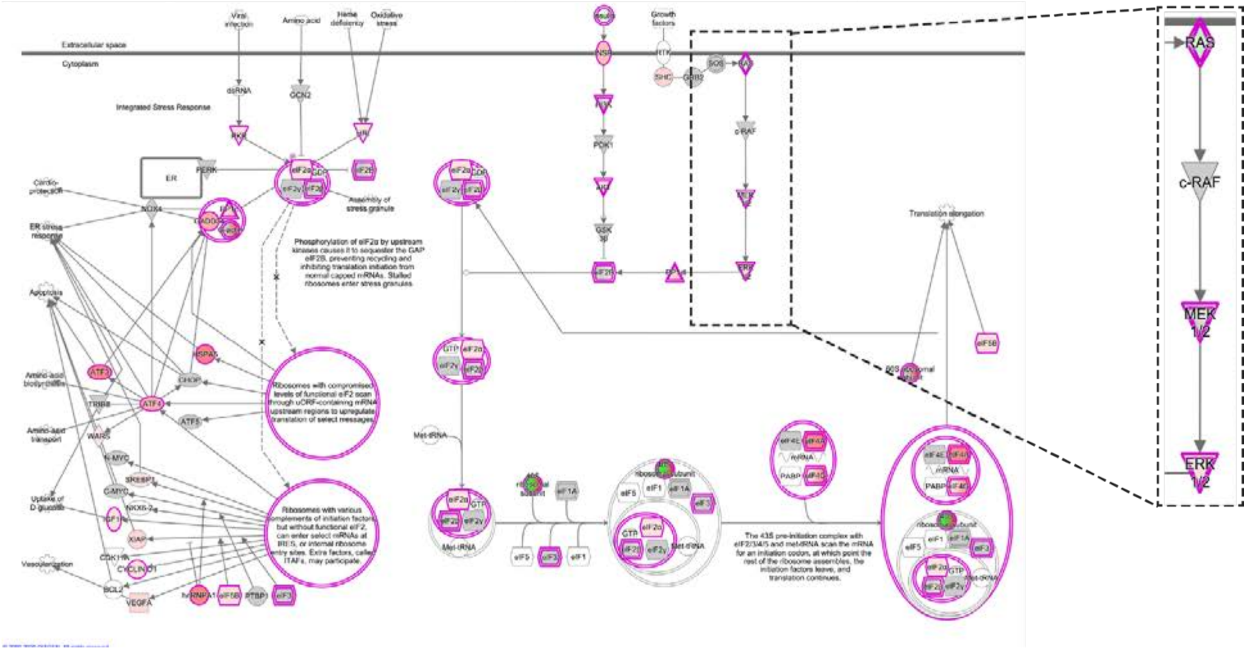

C

Murine mTOR Signaling

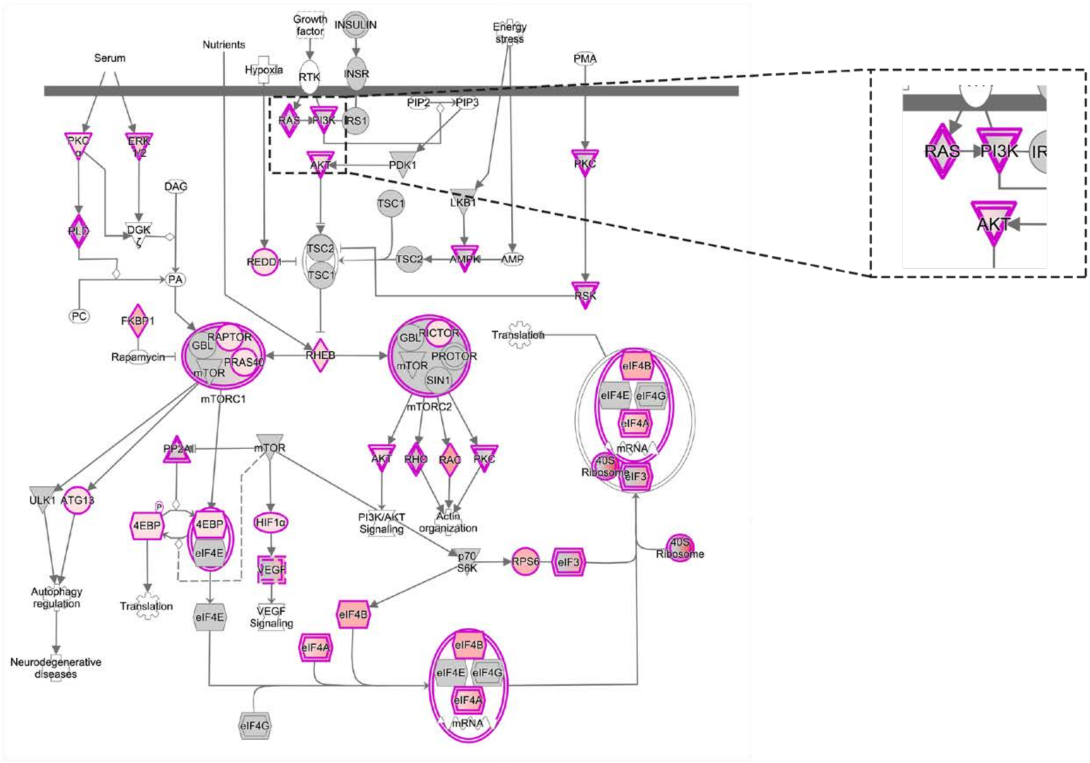

Human mTOR Signaling

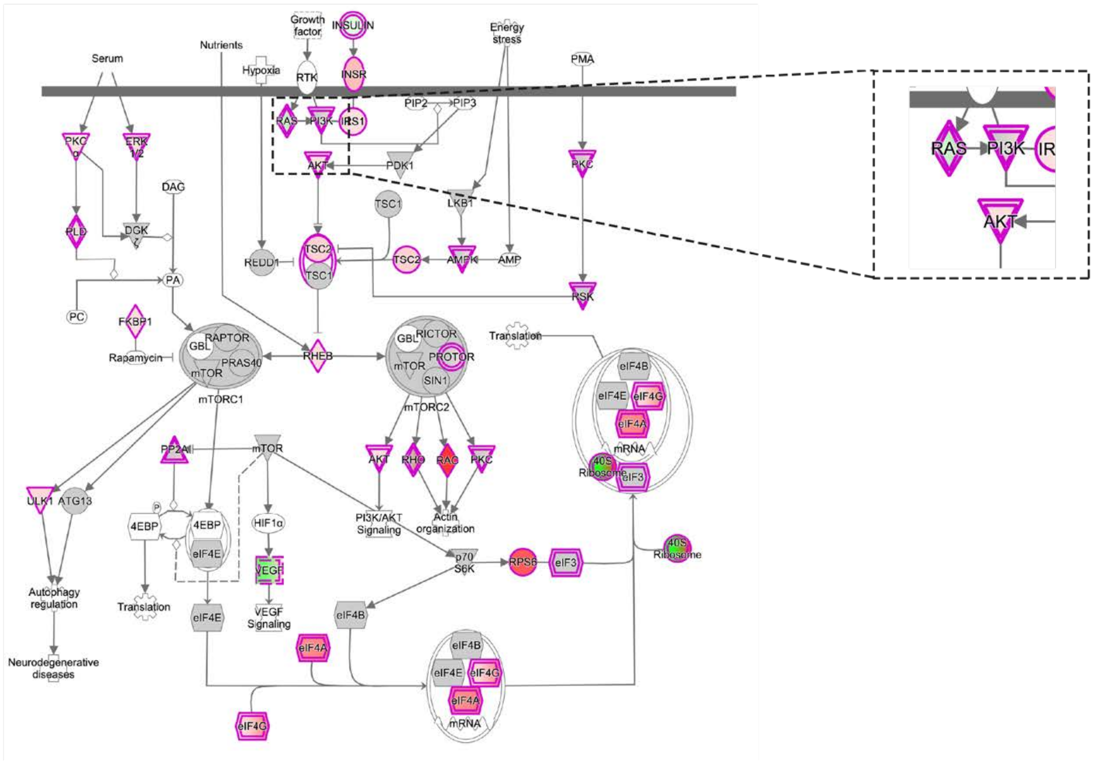

d

Murine eIF4/p7S6K Signaling

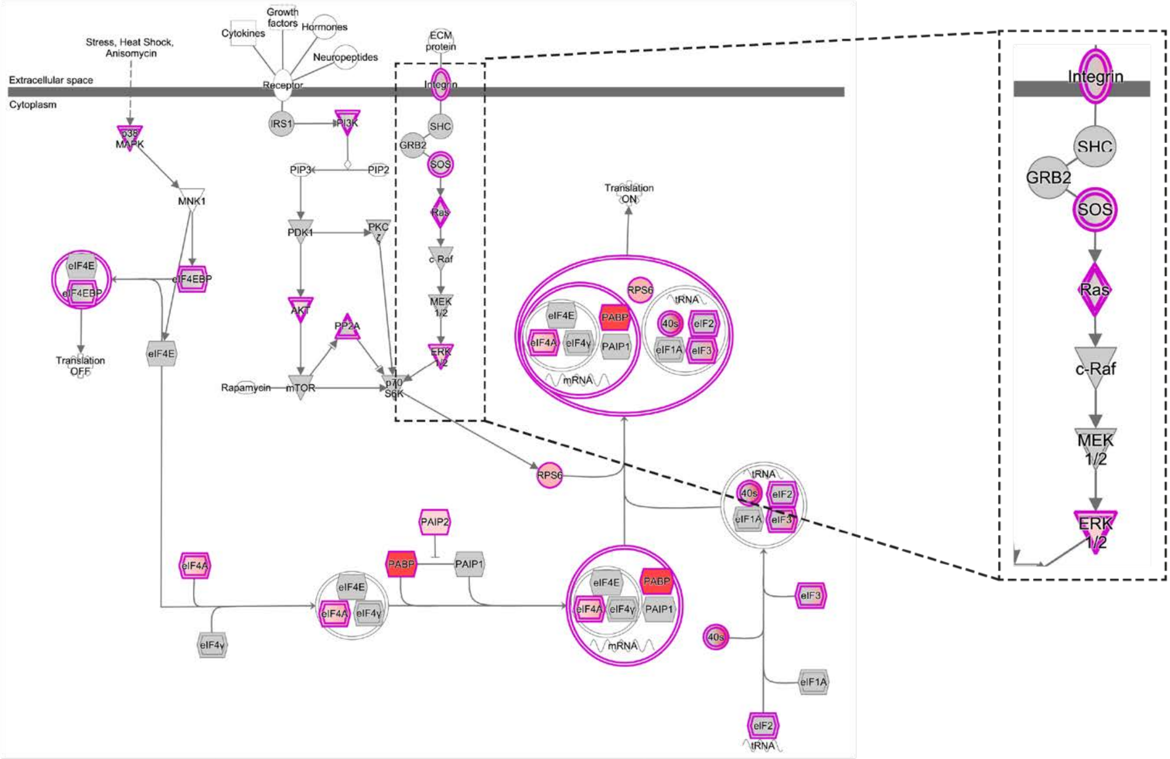

Human eIF4/p7S6K Signaling

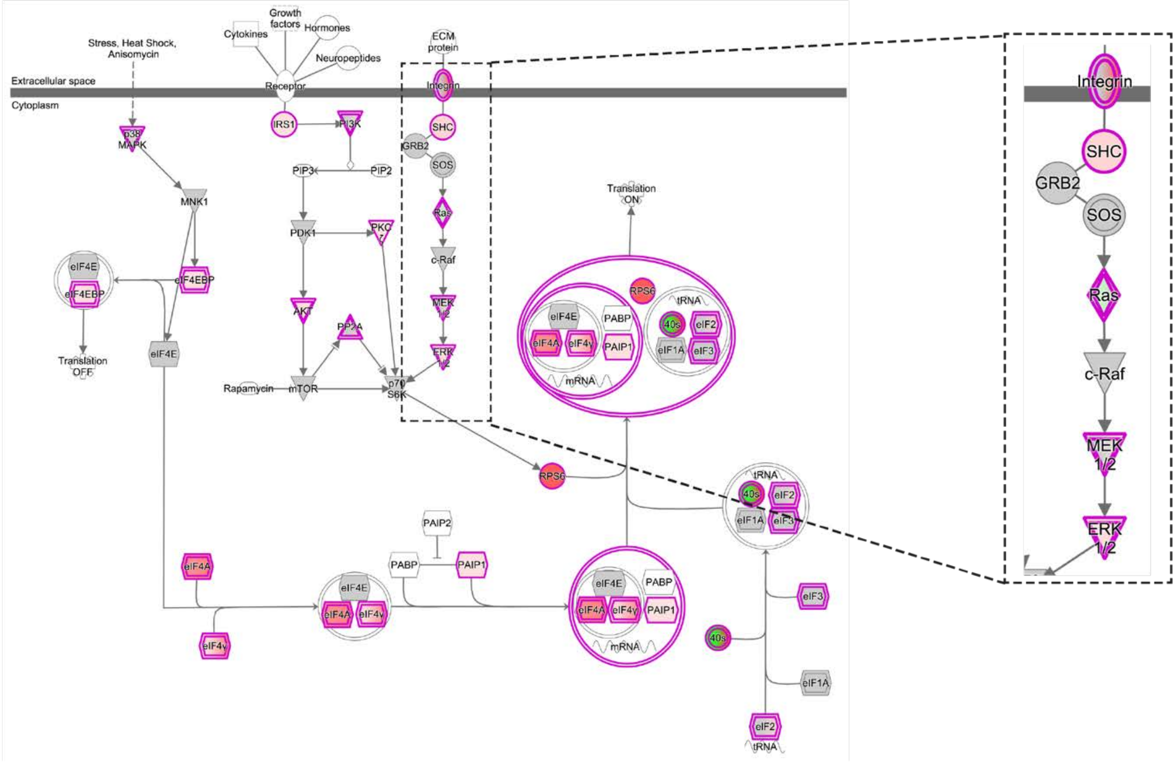

e

Murine Integrin Signaling

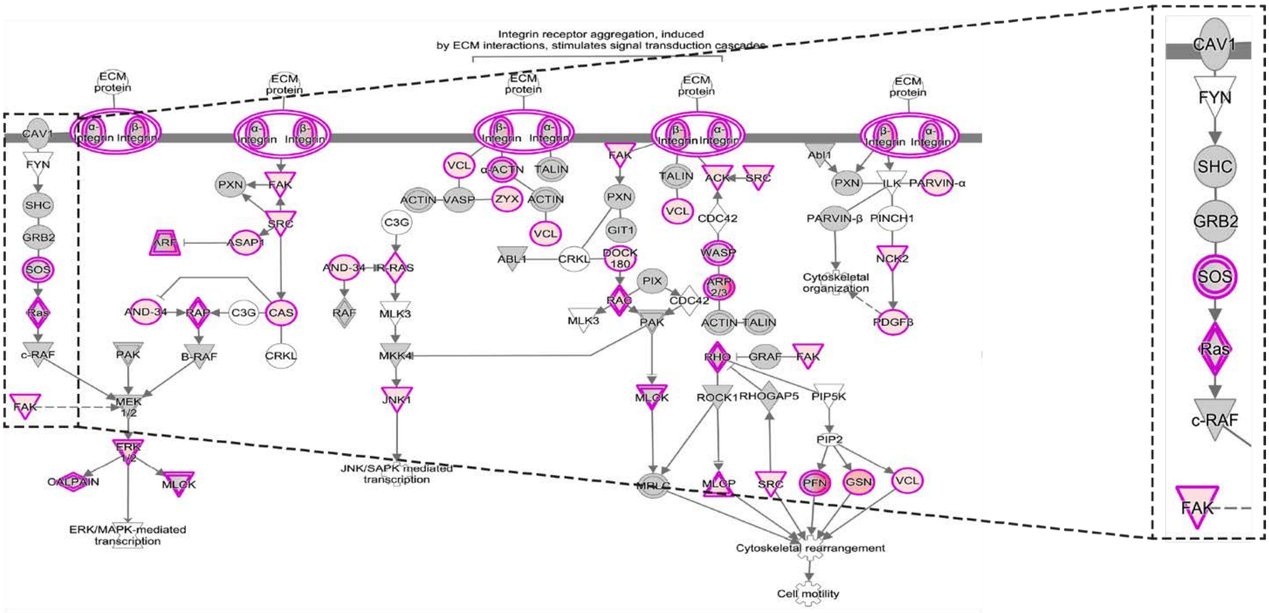

Human Integrin Signaling

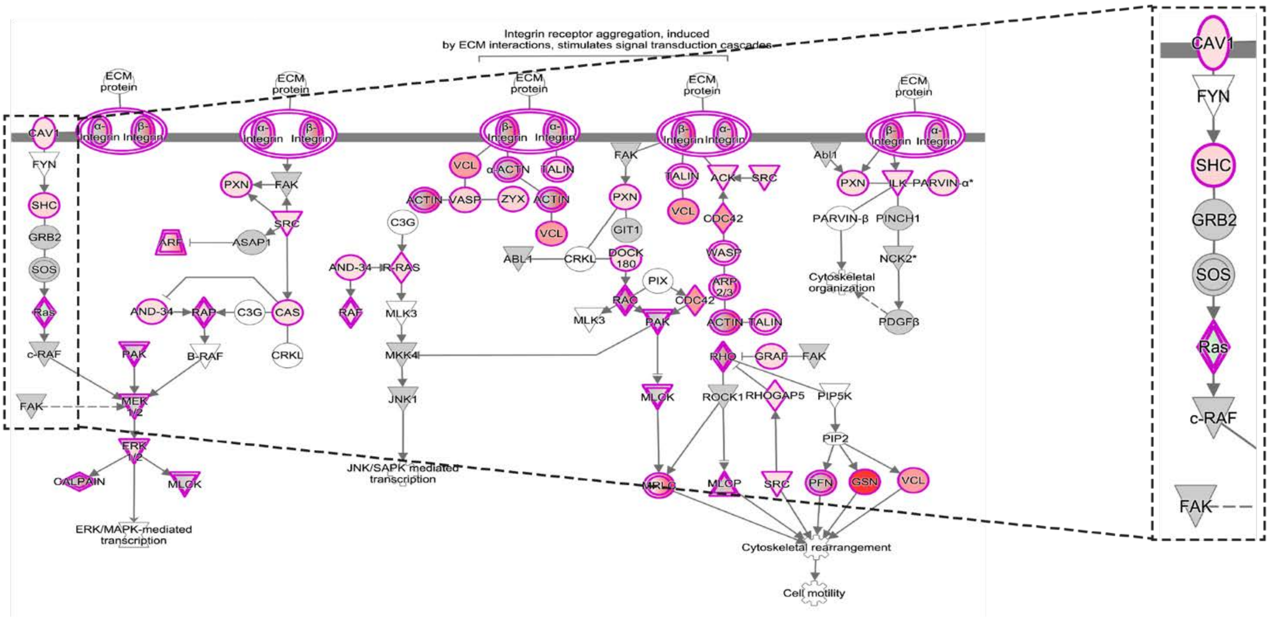

f

Murine C10 Vs Murine Kras Upregulated Genes

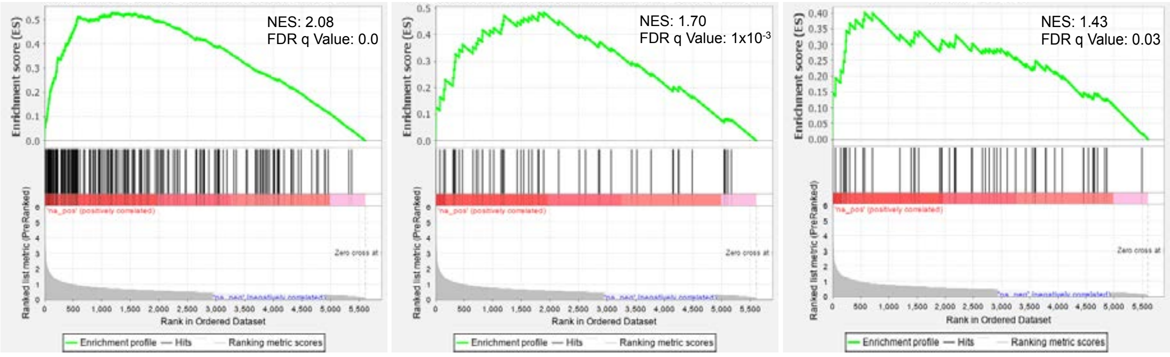

g

Human C10 Vs Human KRAS Upregulated Genes

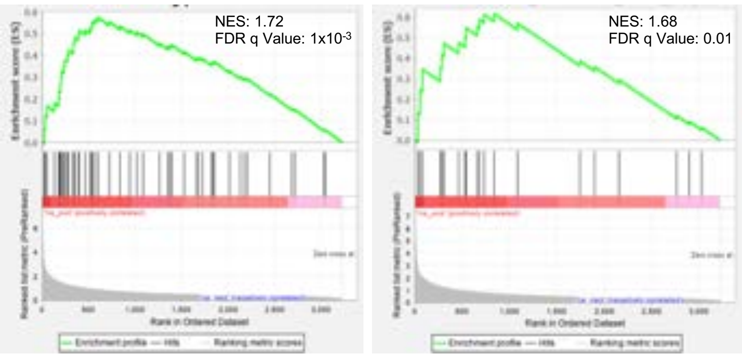

h

Human C10 Vs Overexpressed Genes with Amplified Copy Number in NSCLC

Human C10 Vs EGFR Signaling

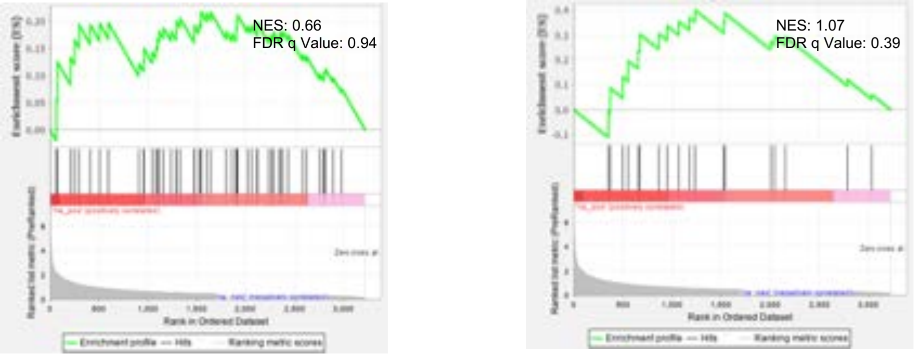

i

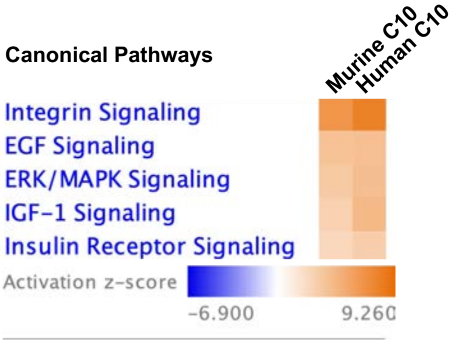

j

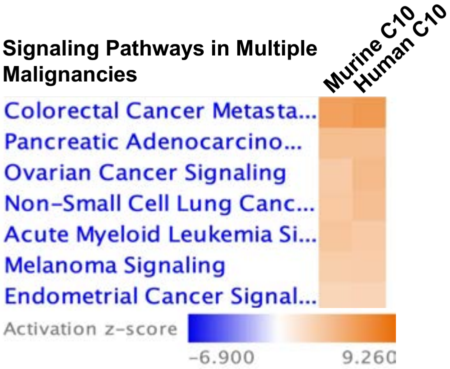

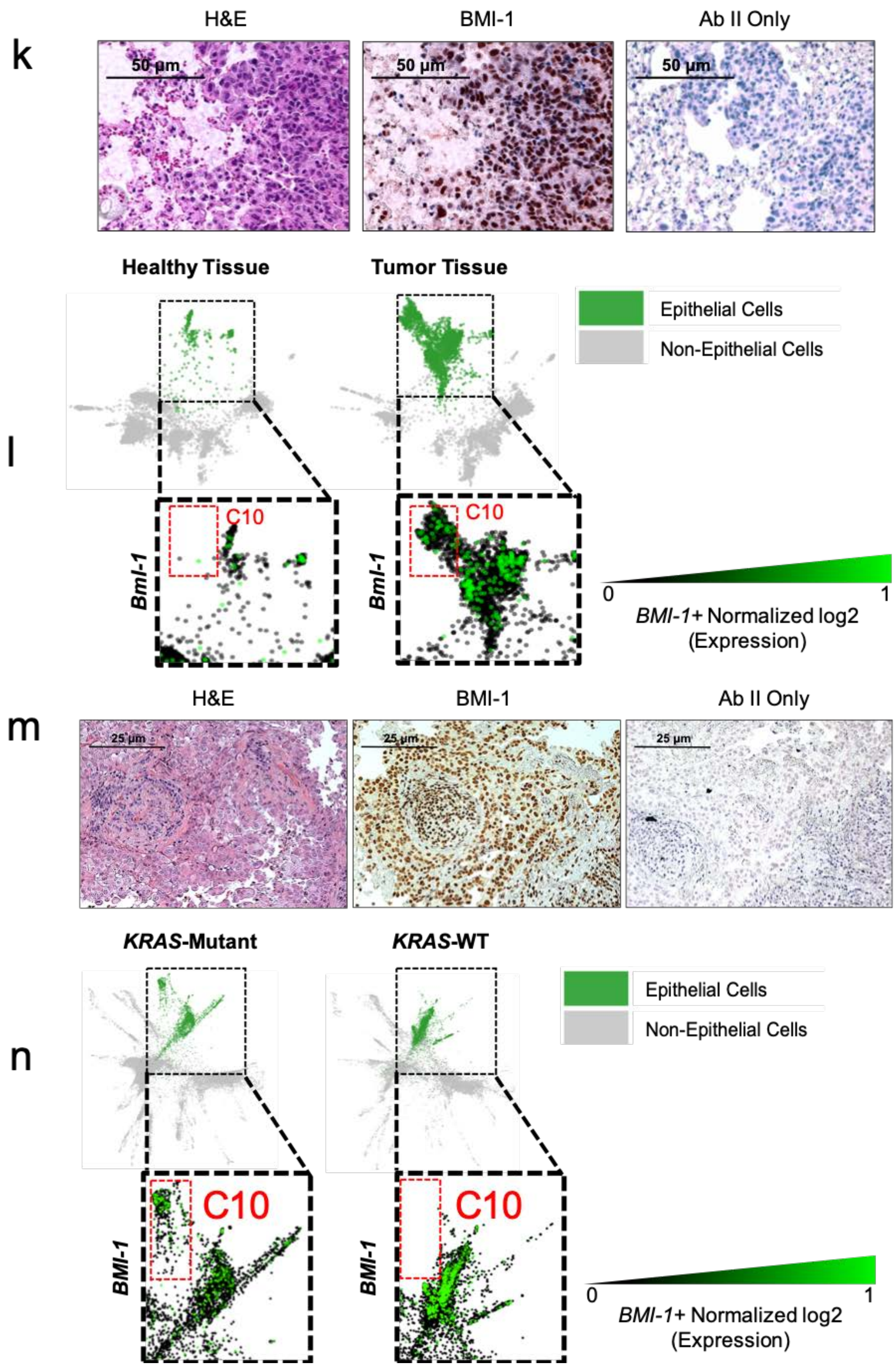
k
H&E
BMI-1
Ab II Only
50 µm
50 µm
50 µm
l
Healthy Tissue
Tumor Tissue
Epithelial Cells
Non-Epithelial Cells
C10
C10
Bml-1
Bml-1
0
1
BMI-1+ Normalized log2 (Expression)
m
H&E
BMI-1
Ab II Only
25 µm
25 µm
25 µm
n
KRAS-Mutant
KRAS-WT
Epithelial Cells
Non-Epithelial Cells
C10
C10
BMI-1
BMI-1
0
1
BMI-1+ Normalized log2 (Expression)

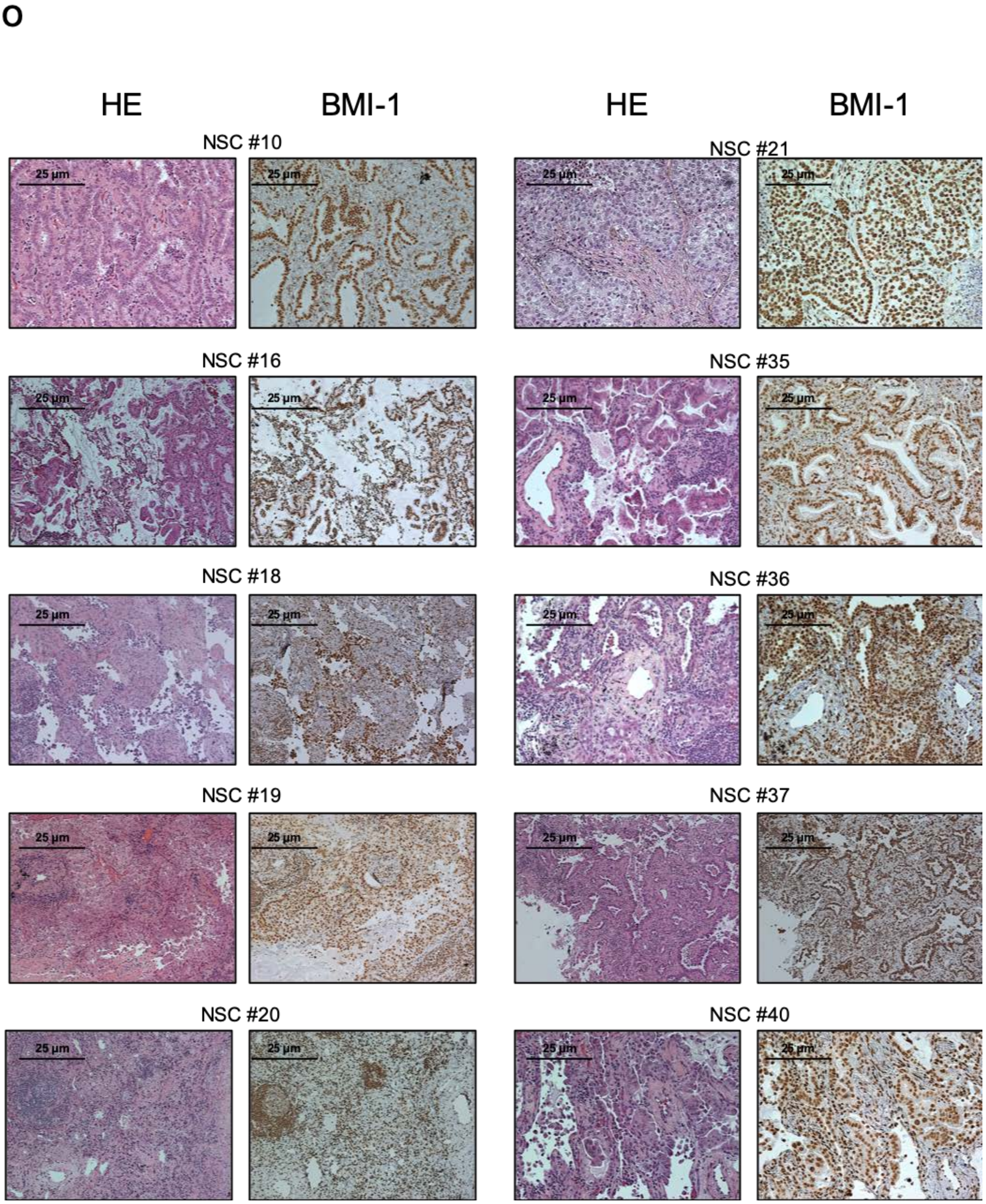
O
HE
BMI-1
HE
BMI-1
NSC #10
NSC #21
NSC #16
NSC #35
NSC #18
NSC #36
NSC #19
NSC #37
NSC #20
NSC #40
25 µm

a SPRING plots, generated only on the adenocarcinoma subset, show the common signature enrichment score for cluster C3 (highlighted by the red dotted box), calculated for each cell equivalent to the number of detected genes from the common signature, as similarly seen for C10. For the positive/upregulated and the negative/downregulated signature the more genes detected per cell, the stronger the enrichment score, represented as a scale from 0 (grey) to 0.5 (blue) to 1 (red), where an enrichment score of 1 signifies detected expression of all marker genes within that cell. **b** EIF2**, c,** mTor**, d** eIF4/p70S6K, **e** Integrin signaling pathways identified by IPA analysis as enriched in both murine and human C10. Pink color indicates activation. Activated KRAS pathway is highlighted by the black dotted squares. GSEA analysis showing comparison of **f** murine and **g** human C10 signature to, respectively, 3 and 2 curated datasets associated to Kras upregulated genes; **h** GSEA analysis showing the comparison between the human C10 signature and 2 curated datsets unrelated to KRAS pathway. **i** Heatmap showing activation z-score of KRAS downstream pathways in murine and human C10s. **j** IPA analysis of murine and human C10 signaling pathways involved in multiple malignancies associated to *KRAS* mutation. **k** Representative pulmonary ADC growing in *KP* mice, stained with hematoxylin-eosin HE (left panel). The middle panel depicts BMI-1 protein expression in the ADC tissue (brown color). The right panel shows staining with secondary antibody only (scale bar 50 µm). **l** Murine TEC-C10 is positive for *Bmi-1* expression in tumor tissue. **m** Representative histological human ADC section stained with HE (left panel). The middle panel shows expression of the oncogene BMI-1 (brown). The right panel shows staining with secondary antibody only (scale bar 25 µm). **n** Human C10 is positive for *BMI-1* expression in *KRAS*-mutant patients. **o** HE and IHC staining for BMI-1 show that all 10 human ADC samples are positive for BMI-1 (scale bar 25 µm).

Supplementary Figure 4. Structure of the compounds.

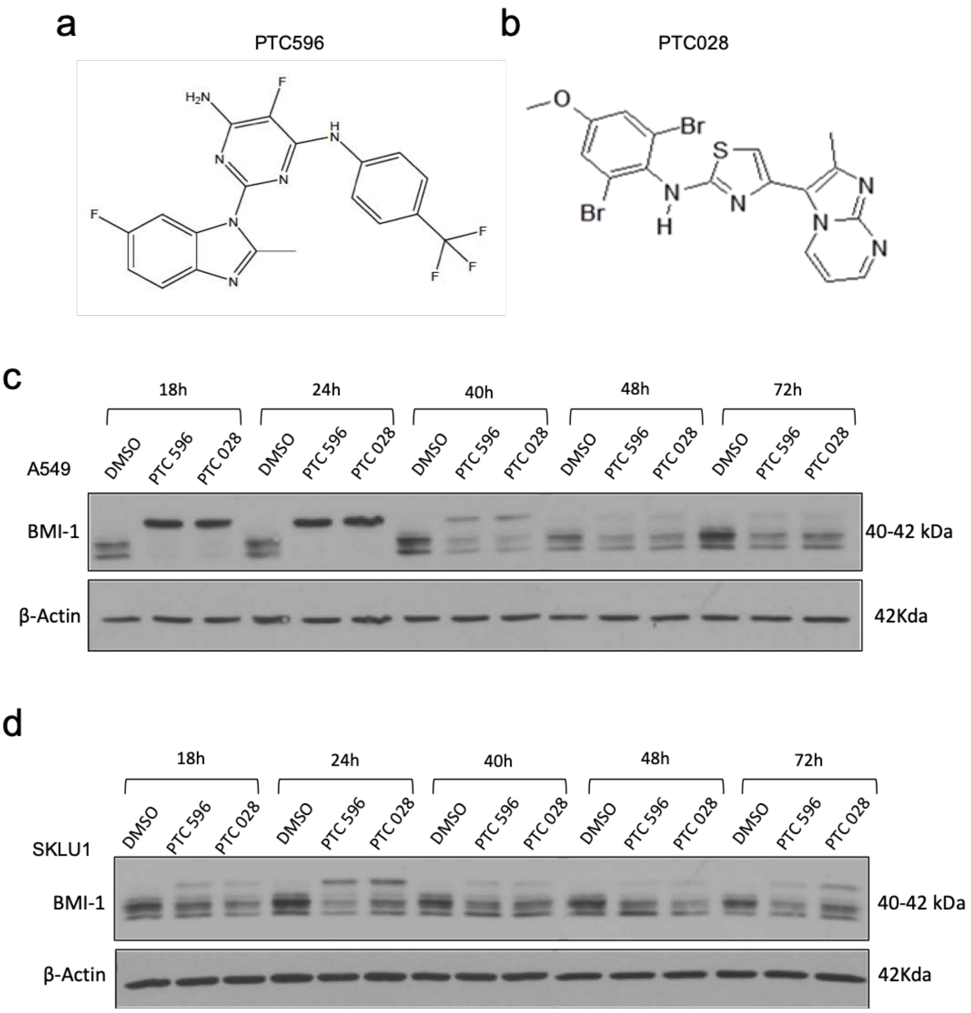

Chemical structures of **a** PTC596 and **b** PTC028 are depicted. Western Blots show BMI-1and β-Actin protein expression in extended time courses (18, 24, 40, 48 and 72 hours) for DMSO, PTC596 and PTC028 of **c** A549 and **d** SKLU1 cells, respectively.

Supplementary Figure 5. Efficacy of PTC596 treatment on KP tumors.

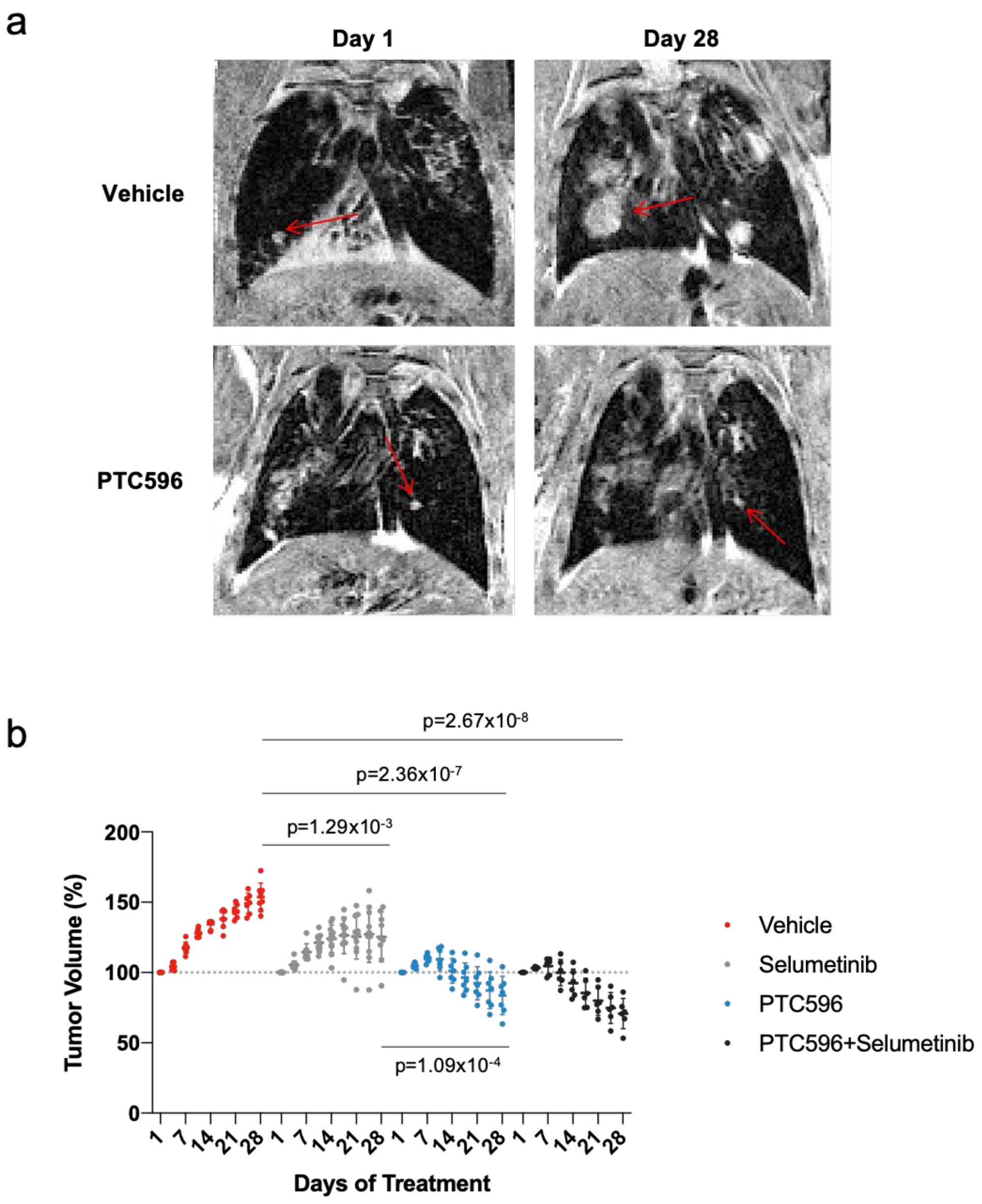

a Representative pictures of tumors (indicated by the arrow), as imaged by MRI in a Vehicle- and a PTC596-treated mouse, at day 1 and 28 of treatment. **b** MRI quantification of KP mice treated for 28 days with Vehicle (n=8, red dots), Selumetinib (n=12, grey dots), PTC596 (n=7, light blue dots) and a combination of PTC596+Selumetinib (n=6, black dots). Bars indicate SD. P values are shown.
