# Supplementary material for: Identification of a targetable KRAS-mutant epithelial population in non-small cell lung cancer
Source: Commun Biol. 2021 Apr 14;4:370. doi: 10.1038/s42003-021-01897-6 (PMC8046784; doi:10.1038/s42003-021-01897-6)
Supplement: Supplementary file 3 — Description of Additional Supplementary Files [file 42003_2021_1897_MOESM3_ESM.pdf]

Description of Additional Supplementary Files

File name: Supplementary Data

Description: Patients' data.

File name: Supplementary Data 2

Description: Source data for graphs:

- Figure 6d
- Figure 6f
- Figure 6h
- Figure 7a
- Figure 7f
- Supplementary Figure 5b
