# Supplementary material for: Identification of a targetable KRAS-mutant epithelial population in non-small cell lung cancer
Source: Commun Biol. 2021 Apr 14;4:370. doi: 10.1038/s42003-021-01897-6 (PMC8046784; doi:10.1038/s42003-021-01897-6)
Supplement: Supplementary file 6 — Reporting Summary [file 42003_2021_1897_MOESM6_ESM.pdf]

nature research

Corresponding author(s): Elena Levantini

Last updated by author(s): Feb 8, 2021

# Reporting Summary

Nature Research wishes to improve the reproducibility of the work that we publish. This form provides structure for consistency and transparency in reporting. For further information on Nature Research policies, see Authors & Referees and the Editorial Policy Checklist.

## Statistics

For all statistical analyses, confirm that the following items are present in the figure legend, table legend, main text, or Methods section.

| n/a | Confirmed | |
|---|---|---|
| ☐ | ☒ | The exact sample size (*n*) for each experimental group/condition, given as a discrete number and unit of measurement |
| ☐ | ☒ | A statement on whether measurements were taken from distinct samples or whether the same sample was measured repeatedly |
| ☐ | ☒ | The statistical test(s) used AND whether they are one- or two-sided<br>*Only common tests should be described solely by name; describe more complex techniques in the Methods section.* |
| ☒ | ☐ | A description of all covariates tested |
| ☒ | ☐ | A description of any assumptions or corrections, such as tests of normality and adjustment for multiple comparisons |
| ☐ | ☒ | A full description of the statistical parameters including central tendency (e.g. means) or other basic estimates (e.g. regression coefficient) AND variation (e.g. standard deviation) or associated estimates of uncertainty (e.g. confidence intervals) |
| ☐ | ☒ | For null hypothesis testing, the test statistic (e.g. *F*, *t*, *r*) with confidence intervals, effect sizes, degrees of freedom and *P* value noted<br>*Give P values as exact values whenever suitable.* |
| ☒ | ☐ | For Bayesian analysis, information on the choice of priors and Markov chain Monte Carlo settings |
| ☒ | ☐ | For hierarchical and complex designs, identification of the appropriate level for tests and full reporting of outcomes |
| ☒ | ☐ | Estimates of effect sizes (e.g. Cohen's *d*, Pearson's *r*), indicating how they were calculated |

*Our web collection on statistics for biologists contains articles on many of the points above.*

## Software and code

Policy information about availability of computer code

| | |
|---|---|
| Data collection | Trimmomatic (v0.36), R (v3.6.2), SPRING (https://github.com/AllonKleinLab/SPRING/) |
| Data analysis | Trimmomatic (v0.36), R (v3.6.2), SPRING (https://github.com/AllonKleinLab/SPRING/), yarr packages (https://github.com/ndphillips/yarr), Ingenuity Pathway Analysis (IPA; Ingenuity® Systems, www.ingenuity.com), GSEA (Broad Institute), TCGA (https://tcga-data.nci.nih.gov/tcga/), EnrichR software analysis (https://amp.pharm.mssm.edu/Enrichr/), PASW Statistics 18 (SPSS Inc.) |

For manuscripts utilizing custom algorithms or software that are central to the research but not yet described in published literature, software must be made available to editors/reviewers. We strongly encourage code deposition in a community repository (e.g. GitHub). See the Nature Research guidelines for submitting code & software for further information.

## Data

Policy information about availability of data

All manuscripts must include a data availability statement. This statement should provide the following information, where applicable:

- Accession codes, unique identifiers, or web links for publicly available datasets
- A list of figures that have associated raw data
- A description of any restrictions on data availability

GEO accession number for Single cell RNA sequencing data (Figure 1, Figure 2, Figure 5): GSE136246 (Currently set to private till August 2020)

# Field-specific reporting

Please select the one below that is the best fit for your research. If you are not sure, read the appropriate sections before making your selection.

☒ Life sciences ☐ Behavioural & social sciences ☐ Ecological, evolutionary & environmental sciences

For a reference copy of the document with all sections, see nature.com/documents/nr-reporting-summary-flat.pdf

# Life sciences study design

All studies must disclose on these points even when the disclosure is negative.

| | |
|---|---|
| Sample size | For single cell RNA sequencing experiments sample-size calculation was not performed. The sample-size of the samples assayed was determined based on availability of clinical samples and mice carrying tumors of similar size. The number of mice required for the MRI experiments depicted in Figure 5 was not based on differences identified a priori, nor were formal power calculations done before conducting those experiments. For cell cycle experiments and immunohistochemistry a biological triplicate approach was adopted. |
| Data exclusions | N/A |
| Replication | All attempts at replication were successful |
| Randomization | For in vivo treatment mice were randomized when tumors reached comparable size |
| Blinding | MRI facility personnel and animal facility personnel measuring tumor size were blind |

# Reporting for specific materials, systems and methods

We require information from authors about some types of materials, experimental systems and methods used in many studies. Here, indicate whether each material, system or method listed is relevant to your study. If you are not sure if a list item applies to your research, read the appropriate section before selecting a response.

## Materials & experimental systems

| n/a | Involved in the study | |
|---|---|---|
| ☐ | ☒ | Antibodies |
| ☐ | ☒ | Eukaryotic cell lines |
| ☒ | ☐ | Palaeontology |
| ☐ | ☒ | Animals and other organisms |
| ☐ | ☒ | Human research participants |
| ☒ | ☐ | Clinical data |

## Methods

| n/a | Involved in the study | |
|---|---|---|
| ☒ | ☐ | ChIP-seq |
| ☐ | ☒ | Flow cytometry |
| ☒ | ☐ | MRI-based neuroimaging |

## Antibodies

| | |
|---|---|
| Antibodies used | anti-BMI-1 ( Cell Signaling Technologies #6964S), anti-β-actin mouse antibody (Santa Cruz #81178), HRP-conjugated secondary antibodies, anti-rabbit IgG-HRP (Santa Cruz #SC2054) or anti-mouse-IgG-HRP (Santa Cruz #SC2031) |
| Validation | anti-BMI-1 ( Cell Signaling Technologies #6964S) has been validated through western blot upon Bmi-1 overexpression and anti-β-actin mouse antibody (Santa Cruz #81178) from Santa Cruz website |

## Eukaryotic cell lines

Policy information about cell lines

| | |
|---|---|
| Cell line source(s) | ATCC |
| Authentication | STR matching analysis attached to the manuscript |
| Mycoplasma contamination | cell lines tested negative |
| Commonly misidentified lines (See ICLAC register) | N/A |

# Animals and other organisms

Policy information about studies involving animals; ARRIVE guidelines recommended for reporting animal research

| | |
|---|---|
| Laboratory animals | K-Ras G12D/p53 null mice (Lox-stop-lox/LSL x K-RasG12D, p53 flox), on average 6-8 months of age and both geneder were similarly distributed. NSG mice were 2-4 months old and gender similarly distributed. |
| Wild animals | N/A |
| Field-collected samples | N/A |
| Ethics oversight | IACUC from Beth Israel Deaconess Medical Center |

Note that full information on the approval of the study protocol must also be provided in the manuscript.

# Human research participants

Policy information about studies involving human research participants

| | |
|---|---|
| Population characteristics | Age, gender and treatment are described in the paper in Supplementary Table 1 |
| Recruitment | This study was conducted with approval of the Dana-Farber Brigham and Women's Cancer Center IRB and written informed consent from subjects. The protocol allows collection of discarded tissue. Human tissue samples were de-identified before transfer to the laboratory, and analysis is not considered human subject research under the US Department of Human and Health Services regulations and related guidance (45 CFR part 46). |
| Ethics oversight | IRB from Brigham and Women Hospital |

Note that full information on the approval of the study protocol must also be provided in the manuscript.

# Flow Cytometry

## Plots

Confirm that:

- [x] The axis labels state the marker and fluorochrome used (e.g. CD4-FITC).
- [x] The axis scales are clearly visible. Include numbers along axes only for bottom left plot of group (a 'group' is an analysis of identical markers).
- [ ] All plots are contour plots with outliers or pseudocolor plots.
- [x] A numerical value for number of cells or percentage (with statistics) is provided.

## Methodology

| | |
|---|---|
| Sample preparation | A combination of Vybrant DyeCycle Violet and Pyronin Y was used for the differential staining of cellular DNA and RNA. A549 cells treated for 24 hours with PTC596, PTC028 and DMSO, were permeabilized in phosphate-citrate buffer solution (pH 4.8), washed in PBS 1x, and then resuspended in a solution of 5 µM Vybrant DyeCycle Violet (Thermofisher Scientific) and 4 µg/ml pyronin Y (Polysciences). Cycle status was then evaluated by flow cytometry. |
| Instrument | Cytoflex Flow Cytometer (Beckman Coulter Inc.) |
| Software | FlowJo |
| Cell population abundance | N/A |
| Gating strategy | Doublets removal as well as Unstained cells and single stained cells for proper gating. |

- [ ] Tick this box to confirm that a figure exemplifying the gating strategy is provided in the Supplementary Information.
